# Applying the principle of justice in digital health

**DOI:** 10.1038/s41746-025-01877-8

**Published:** 2025-07-21

**Authors:** Oriol Yuguero, Pere Ruiz-Trujillo, Montse Esquerda, Nuria Terribas, Marta Aymerich

**Affiliations:** 1 E-Rlab, Ehealth Center, Universitat Oberta de Catalunya (UOC), Barcelona, Spain; 2 Faculty of Medicine, University of Lleida, Lleida, Spain; 3 Sarrià Chemical Institute (IQS), University Ramon Llull, Barcelona, Spain; 4 Global Research on Wellbeing Research Group (GRoW), University Ramon Llull, Barcelona, Spain; 5 Chair in Bioethics Fundació Grífols, Centre for Health and Social Care Research (CESS), Central University of Catalona (UVic-UCC), Vic, Spain

**Keywords:** Health care, Medical research

## Abstract

In an increasingly digital healthcare landscape, it is essential to assess the ethical impact of technology on care practices. We present a conceptual framework on equity in digital health, addressing justice-related concepts and challenges to reducing the digital divide. An ethical approach ensures that digital health respects fundamental rights. We propose goals—such as incorporating digital determinants into public health policy—to promote justice and reduce health inequalities in digital health implementation.

## Introduction

Digital health represents both a transformative opportunity and a profound ethical challenge. As healthcare systems embrace technology, questions about fairness, access, and inclusion become central. This paper examines the digital transformation of healthcare through the lens of bioethics, with a particular focus on the principle of justice. Drawing from philosophical foundations, classical and contemporary theories of justice, and practical examples, we propose a framework to understand and reduce the digital divide. Our goal is to connect ethical theory to actionable strategies that ensure digital health innovations benefit all—especially those most at risk of exclusion.

Healthcare ethics is a way of understanding and practising clinical care based on principles and values. At a time when healthcare is becoming increasingly digitized, we need to think about the possible impact of the use of digital technology and connectivity on day-to-day healthcare practices and, therefore, on healthcare ethics.

The Belmont Report, published in September 1978, defined for the first time the bioethical principles of autonomy, beneficence, and justice. The following year, Beauchamp and Childress published their *Principles of Biomedical Ethics*^1^, which added a fourth principle to the ones presented in the Belmont Report—the principle of non-maleficence (*primum non nocere*). Although these principles are not the only ones to consider when making decisions in bioethics, they are an important and useful tool when analyzing a particular case and can help to prioritize ethical values when they come into conflict. The four principles sum up accurately the commitments that respect for human dignity should entail.

Recently, Sim & Cassel^2^ have advocated the adaptation of the Belmont principles to the new reality, positing that the rapid evolution of Artificial Intelligence (AI) exceeds our ability to develop a traditional ethical consensus in response to it. In this new situation, it seems important to reassess these principles and their application to the entire field of digital health—and not only with respect to AI, which is only a part of it.

The concept of digital health is based on the use of information and communication technologies (ICTs) to achieve health goals. Its success depends on the ability of the public to connect with and gain access to the health services they need.

Digital health represents a transformative paradigm shift in healthcare delivery, defined as the multidisciplinary field leveraging ICTs to enhance health systems, improve healthcare delivery, empower individuals, and advance population health outcomes. Its scope extends beyond narrow technical interpretations to include interoperable data infrastructures, such as electronic health records (EHRs), which enable seamless information exchange across care settings, thereby supporting clinical decision-making and care continuity^3^. According to the World Health Organization (WHO), it encompasses tools such as EHRs, telemedicine, mobile health (mHealth), and AI-enabled solutions that enhance prevention, diagnosis, and management of disease^4^. Advanced analytics and intelligence, such as AI for diagnostic imaging or predictive modeling, leverage big data from wearables, genomics, and social determinants to enable precision health interventions^5^.

The European Commission adds that digital health should ensure equity, interoperability, and systemic transformation of care with active patient participation in health management^6^. This holistic framework, aligned with the WHO’s and European Commission’s definition, positions digital health as an ecosystem that spans prevention, diagnosis, and chronic disease management, while emphasizing equity, interoperability, and system-wide transformation^7^. In this paper, we adopt a broad definition that integrates both perspectives and emphasizes digital health as an ecosystem with ethical and structural implications.

The aim of this Perspective is to explore how contemporary theories of justice can inform ethical and equitable responses to the digital gap in health, with particular emphasis on digital determinants of health (DDH) and strategies to reduce structural exclusion through inclusive design, policy, and institutional innovation.

## Equality, equity, and justice: conceptual clarifications

To anchor the ethical discussion, it is essential to clarify three key terms often used interchangeably but conceptually distinct: equality, equity, and justice. Equality refers to treating everyone the same, regardless of individual differences or needs. Equity recognizes that people have different circumstances and allocates resources and opportunities accordingly, aiming to reach fair outcomes. Justice goes one step further—it involves addressing the root causes of inequality and actively removing structural barriers, ensuring that systems and policies promote fairness for all. and will be revisited later through the adapted Tony Ruth framework.

A major problem in this regard is what is known as the “digital gap,” a phenomenon that has left many people who lack digital skills outside the system. We need to reduce the digital divide by promoting strategies that ensure no one is left behind by digitalization. This requires inclusive policies that consider access, skills, and support. The Digital Health group of the Catalan Society of Family and Community Medicine (CAMFIC) argues that, with the digital transformation of healthcare, digital issues should be addressed alongside traditional social determinants of health^8^.

In this context, the concept of *digital determinants of health* has emerged as a critical framework for understanding how digital environments shape health outcomes and access. These determinants include factors such as access to digital infrastructure, digital literacy, cultural and linguistic inclusion, algorithmic bias, data governance, and trust in technology. As Chidambaram^9^ argues, DDH intersect with traditional social determinants such as education, socioeconomic status, and geography, and may even amplify existing disparities if not properly addressed. Incorporating this framework allows for a more nuanced analysis of the digital gap and reinforces the ethical imperative of equity in digital health transformation.

## Bioethical principles and their relevance

The four fundamental principles of bioethics, as articulated by Beauchamp and Childress^1^, are: autonomy (respecting individuals’ capacity to make informed choices), beneficence (promoting well-being), non-maleficence (avoiding harm), and justice (ensuring fairness in the distribution of benefits and burdens). These principles serve as an ethical framework to guide clinical decision-making and health policy.

In the context of digital health, these principles take on new dimensions. Autonomy involves informed consent for the use of digital tools, data sharing, and AI-supported care. Beneficence includes the use of technology to improve outcomes, such as through telemedicine or personalized interventions. Non-maleficence requires addressing risks such as data breaches, algorithmic errors, or increased health disparities. Justice, the central focus of this article, is a principle we will explore in greater depth through its practical implications.

## Philosophical foundations of justice

Justice is one of a group of concepts that are generically termed “philosophical.” This is a label applied (with a certain disdain by some people) to ideas that are difficult to define, as if this difficulty were reason enough for us to ignore them. However, in clinical practice and specifically also in digital health, they are especially important.

Ever since human beings began to think in a methodical way, we have thought about “philosophical” ideas, such as the ideas of what is good or what is just. Many of the assumptions that form the basis of our thinking on these issues in the West can be traced back to Plato and Aristotle. Plato speaks of justice in the important dialog *The Republic*^10^, in which, both for individuals and for societies, justice is related to the idea that everything is in its proper place.

For his part, Aristotle presents justice as a way of being, which consists of doing and wanting what is fair^11^, and identifies different kinds of justice. In distributive justice, for instance, what is fair is a proportionate distribution of goods and honors. In corrective justice, it is the punishment for unjust actions. Always, as is usual in Aristotle, virtue lies in the middle point between the vices of excess and defect.

## Rawls’ theory of justice

Having located the origin of the idea of justice in our culture, we now jump forward in time to the most influential author in contemporary thought on the subject. One cannot talk about the principle of justice without considering A Theory of Justice^12^, by John Rawls. Below, we present a brief outline of the main ideas of the theory and then see how they can be related to the principle of justice in bioethics, and specifically to its application in the field of digital health.

Rawls’s contribution basically consists of generalizing a theory of the social contract already present in authors such as Locke, Rousseau, and Kant and taking their contractualist approach to a higher level of abstraction^12^. This new contractualist theory of justice has turned out to be so influential that much of the subsequent philosophical debate on the concept of justice has in some way been linked to it.

For Rawls, justice is the prime virtue of social institutions, with a status comparable to that of truth in systems of thought^12^. It is interesting to note that his theory openly attacks a utilitarianism that, according to him, has long been the prevailing theory as the foundation of justice. We should stress that he is not looking for a theory of justice aimed at guiding concrete actions, but rather a kind of instruction manual, a heuristic that can help us in our attempts to attain a just society. What is needed, ultimately, is a set of principles for assigning basic rights and duties and ensuring a fair distribution of the burdens and benefits of living in society.

In contrast with utilitarianism, Rawls’ theory presents a deontological approach in which justice, rather than good, must be the basis of social institutions^12^. To put it very simply, for Rawls, the utilitarian principle that maximizes the benefit of the greatest number does not respond completely to our intuitions about what is just. One of the problems is that the kind of arithmetical calculation of costs and benefits that utilitarianism proposes as the foundation of justice falls short.

Another point to bear in mind is that contemporary societies have a high degree of moral, religious, and cultural diversity, and this entails the coexistence of different conceptions of good and justice. In this regard, Rawls starts from the statement that, even setting out from different conceptions of justice, it is possible to agree that social institutions are just when they do not make arbitrary distinctions when assigning rights and basic duties to people and when their rules promote a balance between people competing for the good things in life in each society^12^.

Like the utilitarian standpoint, Rawls sees society members as rational beings (they have the capacity to choose their ends, develop a conception of good, and make decisions about the means to maximize their chances of achieving their goals)^12^. However, for him, besides rationality, human beings also share reasonability; that is to say, the capacity to develop a sense of justice that sometimes makes them act in ways that, at the outset, do not merely seek individual benefit, but accepting and respecting normative principles. So this is our starting point: we must consider that society is made up of rational, reasonable individuals^13^.

Rawls proposes, then, to reach the principles of justice starting from what he calls the “original position,” which is equivalent to the state of nature in the traditional theories of the social contract: an exercise of the imagination which prepares us to think and “sign” the contract on which the just society is to be based. In this original position, individuals live behind the “veil of ignorance,” another of Rawls’s key concepts^12^. Behind this veil, no one knows their place in society, what their natural abilities are, or what their conception of good is. In this state, our ignorance of our socioeconomic status, our sexual identity, our race, our origin, our ideology or our state of health—that is, the contingencies that affect the definition of our particular interests—means that our choice of principles of justice does not seek our individual benefit, simply because we do not know what can benefit us and what can harm us. This veil makes us ignorant of all arbitrary matters; in the original position, on the other hand, we do have knowledge about what the world is like and how it works: we know, for example, that there are social classes, races, sexes, that there are different religious convictions and ideologies, different conceptions of good and justice. And we also have an understanding of how areas such as health or the economy work. In this way, it can be said that we are in an ideal initial situation to agree on a set of fair principles based on impartiality: hence the definition of justice as “impartiality” or “fairness” that Rawls chooses.

According to Rawls, through the process he calls a “reflective equilibrium,” we would eventually obtain two principles of justice^12^. The first, called the equality principle, states that there must be equality when distributing basic rights and duties. The second, known as the difference principle, foresees the possibility of the appearance of social and economic inequalities, but, in order for these to be fair, the principle requires that they produce benefits for everyone and, in particular, for the less favored members of society. These are two manifestations which, according to him, are a special case of a more general conception of justice and which he expresses as follows:

“All social values—liberty and opportunity, income and wealth, and the bases of self-respect—are to be distributed equally unless an unequal distribution of any, or all, of these values is to everyone’s advantage”^14^.

## Other philosophical views of Rawls’s theory

Several criticisms have been directed at Rawls’s theory of justice. Beyond his well-known debate with Nozick—who defended individual rights and a conception of justice based on voluntary transactions without state interference—four key authors offer notable critiques and extensions.

Michael Sandel^15^ challenges Rawls’s theory of distributive justice, arguing that it is difficult to implement without a strong sense of community. He sees justice, in Rawls’s framework, as a remedial virtue that compensates for the absence of other spontaneous virtues such as solidarity and fraternity. Sandel emphasizes the role of communal ties and the common good as essential elements for a more complete conception of justice.

Amartya Sen^16^ introduces a capabilities-based approach, criticizing Rawls’s focus on the distribution of primary goods as overly abstract and insufficient for addressing real-world inequalities. Sen argues that justice should be concerned with individuals’ actual capabilities to achieve well-being, taking into account the diverse ways in which people convert resources into valuable life outcomes.

Norman Daniels^17^, a student of Rawls, applies Rawlsian principles to the right to health. He argues that health is central to ensuring fair equality of opportunity, especially for individuals with illnesses or disabilities. While initially treating health as a primary good, Daniels shifts his approach to avoid conceptual overload. He highlights healthcare’s special role in maintaining normal functioning and protecting the full range of social opportunities.

Ronald Dworkin^18^ critiques the division between civil and social rights, asserting that both are integral to a humanist and democratic society. He argues that liberty and distributive justice must coexist, as justice is fundamentally a matter of resource distribution. For Dworkin, the right to health is part of the basic structure of society and essential for ensuring equal opportunity regardless of economic status.

From this deontological perspective, justice is the foremost virtue of social institutions, taking precedence over conceptions of the good. Within bioethics, this translates into a requirement to establish fair rules that ensure equitable distribution of benefits and burdens, consistent with the principle of equality. Inequalities are only justifiable if they improve the situation of all, especially the least advantaged, in line with Rawls’s difference principle^12^.

These philosophical considerations are not merely theoretical. Across various health systems, structural manifestations of digital injustice are well documented. For instance, telemedicine deployment during the COVID-19 pandemic revealed stark disparities in rural regions, where broadband access was limited and digital literacy among older populations was low^19^. Persons with disabilities often face inaccessible user interfaces, a lack of screen-reader compatibility, or poor mobile design, which further marginalizes them from digital health services^20^. In some cities, “digital redlining” has been observed, where low-income neighborhoods receive poorer digital infrastructure or targeted exclusion from digital innovation programs^21^. Furthermore, algorithmic bias in AI-driven triage or diagnostics has been shown to systematically disadvantage racialized and socioeconomically deprived populations^22^.

These examples illustrate how ethical theories of justice—including Rawls’s difference principle—can inform digital health policies that proactively identify and correct systemic sources of digital exclusion.

## From principles to practice

Translating these ethical foundations into practice requires a structured lens through which to evaluate digital health challenges. Drawing on the principle of justice, we propose an adapted framework to assess digital health interventions not only in terms of access and usability, but also in how they address systemic disparities and promote inclusive innovation.

Justice in healthcare is the expression of the idea of equal opportunities and the application of the difference principle when resources are scarce. A process of digital transformation that understands and recognizes the limitations of a large part of the population would not endanger the principle of justice. The speed of this transformation of the system towards digitization has a huge impact not only on users of healthcare services but on healthcare practitioners as well.

Given that health policies are habitually based on distributive justice, we believe it is important to apply this approach in the field of digital health. When we talk about bioethics and digital health, the principle of justice should lead us to uphold the principle of equal opportunities in the distribution of the benefits and burdens that technological advances bring to society. But to properly address justice, it is not enough to follow the equality principle, ensuring full access for everyone to digital health-related devices, procedures, and systems. In the digital transformation, some inequality is inevitable in favor of those who can adapt more quickly to constant change (and this rapid adaptation may even favor the transformation and its benefits). But the principle of justice must follow as well the difference principle, which requires us to guarantee that these changes also, and especially, benefit “the most disadvantaged,” that is, the people for whom this process of adaptation represents a real challenge.

It is one thing to make digital devices available to the population, and another to ensure that they reap the benefits of the digital transformation. It is important to focus on the obstacles posed by an increasingly technological environment that places certain users at a significant disadvantage, and to try to redress a situation that we would be right to consider an injustice.

In this context, then, justice is the provision of equal, equitable, and appropriate treatment. Anyone who has a valid requirement is entitled to receive this treatment. In contrast, an injustice is a wrongful act or an omission that denies people the benefits to which they have a right, or that fails to distribute the burdens fairly.

**Fig. 1:**
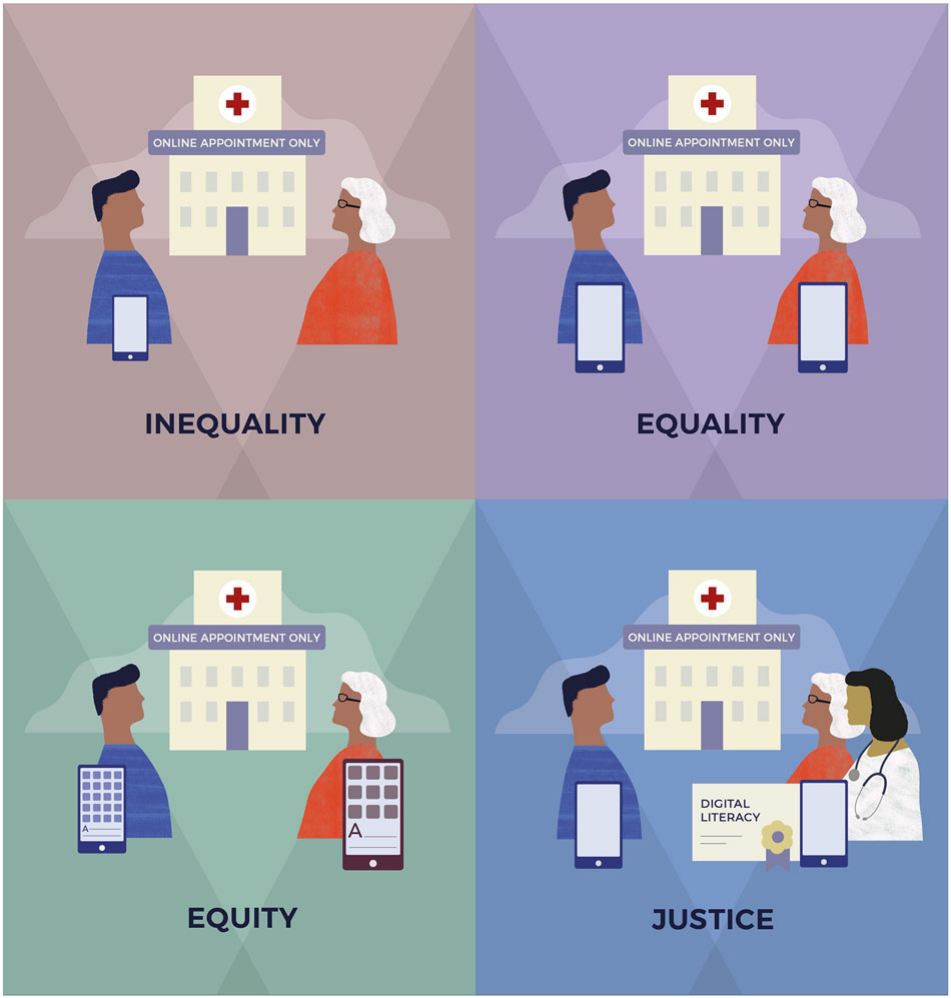
From inequality to justice in digital health. Illustrative model adapted from public health equity frameworks, applied to digital access and inclusion. The figure contrasts different levels of resource distribution and structural response: inequality (no access), equality (same access regardless of need), equity (access adapted to individual needs), and justice (removal of structural barriers and active facilitation of inclusion).

We decided to adapt Tony Ruth’s image^23^ of equity in digital health and the different concepts linked to the principle of justice in order to highlight the challenges we must address if we genuinely intend to reduce the digital gap (Fig. 1).

According to this adapted image, and drawing on the philosophical concepts of justice, equality, and equity introduced earlier, we illustrate how these principles can be interpreted in the field of digital health:

*Inequality*: This refers to situations in which one person has access to an internet connection and an active device, while another does not. In the context of digital health, such inequality is particularly relevant in systems that increasingly promote virtual appointments and provide many services through mobile applications and digital platforms. Without adequate support, this shift may exclude large segments of the population who lack digital access or skills, thereby undermining equitable access to healthcare.

*Equality*: In this scenario, two individuals are provided with the same digital device and internet connection. While this appears to represent equal treatment, it fails to account for specific needs. For example, a person with limited digital literacy or a visual impairment may not be able to use the device effectively, while someone with greater technological competence might find the same resources insufficient. This illustrates how formal equality alone can reproduce or exacerbate disparities.

*Equity*: Equity involves recognizing differences in individuals’ capabilities and contexts, and taking proactive steps to ensure meaningful access. In digital health, this could mean providing a larger device with enhanced visibility for a user with low vision, or customized support for someone with limited technological familiarity. Equity requires tailoring resources to actual needs, rather than assuming a one-size-fits-all solution.

*Justice*: Justice goes beyond individual accommodations and involves structural action to eliminate the root causes of inequality and digital exclusion. This includes investing in digital literacy programs for vulnerable populations and appointing digital health facilitators to assist patients in navigating digital tools and accessing the full range of services available. Such measures aim to create a more just and inclusive digital health ecosystem where everyone can participate and benefit.

We believe that this new image responds to a new vision of justice and digital health. In an article published in 2024^24^, we talked about the significant socioeconomic difference between people who have access to new technologies and the skills needed to use them and those people who do not (the digital gap).

However, the digital gap extends far beyond binary access disparities, functioning as a multilayered determinant of health outcomes shaped by intersecting structural, cultural, and technical forces. Contemporary research conceptualizes this divide through the lens of DDH^25^, which operates across individual, interpersonal, community, and societal levels^26^.

These complexities are formalized in frameworks like the Digital Healthcare Equity Framework, which integrates equity assessments across five lifecycle phases such as planning, development, acquisition, implementation, monitoring^27^. Its core innovation lies in binding *technical design* (e.g., algorithmic audits for bias), *community co-creation* (e.g., participatory prototyping with marginalized users), and *systemic enablers* (e.g., policy mandates for interoperability). From an ethical standpoint, failing to recognize these layered determinants risks reinforcing structural injustice under the guise of digital innovation. Furthermore, digital exclusion is often compounded by mistrust in institutions, especially among communities historically marginalized or subject to surveillance, making trust-building a critical component of equitable design.

Broadly speaking, the general public has a positive impression of digital health^28–30^, but only a very small group derives maximum benefit from the possibilities it offers^3,31^. A review published in 2023^32^ stressed that promoting digital literacy was fundamental to the attempts to encourage the general public to participate actively in decision-making and to pay more attention to their health.

Seeking ways to close the digital gap is one of the main challenges^33^ facing digital health in the short term. For the most part, it is the over-70 age group that is least likely to possess the technological skills needed or be acquainted with electronic tools and devices, since only 28.5% of individuals aged 65–74 in the EU have basic or higher digital skills, such as the ability to use devices like smartphones or computers^34^. This level drops dramatically beyond age 75, being just 9.8% in Spain or 4.6% in Italy^34^. Moreover, a 2023 study of diabetes mellitus patients aged over 65 showed that 85% found digital tools useful, but that no more than 35% actually used them^35^.

These data highlight the need to address this distance so that digitization does not become a barrier to access to the healthcare system. It will be necessary to think about the situations in which people are most vulnerable (due to the lack of training or the absence of an adequate connection) in order to be able to offer real solutions. The new technologies allow people to consult health practitioners rapidly and directly without the need to come to the health center; this capability is in itself a form of justice, because it provides equal opportunities for patients to receive treatment from health professionals when in other situations it would be very difficult.

The current social and demographic reality is unlikely to change in the coming years. In fact, we believe that it will remain largely as it is, because inequalities will not suddenly disappear, and the trend towards greater longevity is certain to continue. The elderly and the more vulnerable members of the population are generally the main users of the health system and at the same time the ones who are most likely to be excluded by digitization; in contrast, younger sectors of the population or those who run no risk of social exclusion do not face this problem because either they are already “digital natives” or they have digitized rapidly in recent years.

Many population groups—including older adults, individuals with disabilities, people with low digital literacy, language minorities, and those with limited socioeconomic resources—are among the main users of healthcare services and at higher risk of exclusion in the digital transformation. Digital health policies must ensure that these communities are not left behind. This is why it is important to involve them in the design of these digital policies or, at the very least, to take their needs into account when planning the digitization of the new digital health system. The attempts to make access to the digital system more equitable must bear in mind the opinions, experiences, and real needs of this group of people in their day-to-day relations with the health services.

The COVID-19 pandemic made this reality clear, imposing digitization in most areas of interaction between government bodies and the public, and perhaps in the health sector more than anywhere else. The pandemic limited face-to-face access and ushered in alternative modes of delivery of healthcare services such as online appointments, virtual consultations, and telemedicine. All of these services offer significant organizational or management advantages, but not everyone will see these changes as beneficial. In this situation, the principle of justice may be seriously undermined.

Digitization poses the challenge of reassessing the scope of ethics in health. Dilemmas that it raises include the appropriate management of large volumes of health data, the privacy of professionals and patients, and the guaranteeing of equitable access to advanced technologies and virtual resources.

## Digital health literacy as an enabler of justice

Digital health literacy should be understood as a critical enabler within a broader set of structural and systemic factors. It plays a central role in empowering individuals, yet must be addressed alongside other DDH—such as infrastructure, accessibility, algorithmic fairness, and trust in institutions—to promote true equity.

The WHO European Region’s Digital Health Action Plan for 2023–2030 identifies enhancing digital literacy and building capacity across the general population as a key regional priority, with special emphasis on training healthcare workers to effectively use digital health services for both disease prevention and treatment^36^.

An ethical perspective in digital health ensures that technological advances and digital interaction are managed respecting fundamental principles and rights—both of those employed by the health system and of those who receive its services. To ensure that the implementation of digital health applies the principle of justice described here, and taking into account the reality of the European Health Systems, we propose the following goals:

- *To establish equitable access to devices and connectivity, providing resources adapted to the specific needs of users*.
  Actions towards this goal could be, under *CLEARS Vulnerability Framework* (addresses Culture, Limiting conditions, Education, Age, Residence, and Socioeconomic status), provide devices with larger screens or voice navigation, or even software that allows content to be enlarged for people with impaired vision. In addition, the Administration could subsidize broadband access, prioritizing the so-called “digital deserts” (areas with no public access to free WiFi, e.g., areas with no public transport or libraries around).
- *To create the figure of a “digital facilitator” in health systems to help people navigate and use the digital resources available*.
  An example of the implementation of this figure can be found in the UK National Health Service^37^. This eHealth facilitator can do the technical setup (installing/configuring apps and devices, troubleshooting connectivity), user education (training patients on app functions or telehealth visit protocol), navigation support (guiding users through portal menus and workflows), and ongoing troubleshooting (Offering follow-up support across the digital health journey).
- *To actively involve members of the public, especially the most digitally vulnerable groups, in the design of digital health policies and solutions through co-creation processes*.
  Engaging users—especially those from underserved backgrounds—through co-creation and participatory design, besides promoting social justice by proactively addressing social determinants and inclusion barriers, can ensure that digital tools are accessible, tailored, and trusted^38^ and can enhance engagement, particularly among older adults, non-native speakers, or low-literacy populations^39^.
  The ProPacient Decalogue, built by end-users themselves within the Catalan Health System, is an example of integrating patient voices in digital health research design^40^.
- *To carry out equity and justice impact assessments before implementing new technologies or digital services in health, as well as to establish mechanisms for continuous monitoring and evaluation of the use and impact of digital technologies in health, with a special focus on the detection and correction of possible inequalities*.
  Equity frameworks can be used for pre-implementation, monitoring, evaluation, and impact of digital health, such as the Digital Healthcare Equity Framework^27^, or the Health Equity Impact Assessment (HEIA) and the Health Equity Assessment Toolkit (HEAT). HEIA is a decision support tool that shows users how a new program, policy, or innovation affects health equity in different population groups^41^. HEAT is a software developed by the WHO to facilitate the assessment of within-country health inequalities^42^.
- *To implement training programmes in digital skills and digital health based on ethical principles, aimed a) at digitally vulnerable groups and b) at health professionals*.
  The WHO European Region’s Digital Health Action Plan for 2023–2030 identifies enhancing digital literacy and building capacity across the general population as a key regional priority, with special emphasis on training healthcare workers to effectively use digital health services for both disease prevention and treatment^36^. Moreover, recent literature reviews emphasize that ethically grounded training programs, both for digitally marginalized individuals^43^ and healthcare professionals^44,45^, are essential to promote digital inclusion, privacy safeguards, and equitable care outcomes.
- *In the design of public health policies, to include digital determinants alongside traditional social determinants as factors that impact health inequality*.
  Actually, in a WHO review, 127 health determinants were found to have emerged or changed during the digital transformation of society, being 37 digital, 33 social, 33 commercial and economic, and 24 political determinants^25^. Without an understanding of the DDH in context, public health implementation may result in tools and knowledge that are incomplete, as they do not address the cumulative or interactive effects of multiple domains^26^. Furthermore, there is already existing literature on how to include the DDH within health professional education^46^.
- *To promote collaboration with other sectors (education, technology, social services) to comprehensively address the factors that define the digital divide in health*.

An example of this cross-sectoral collaboration can be found in the North of England^47^. This research showed that building multi-partner coalitions offering basic digital training in local venues to help residents navigate online health services improved social and emotional resilience.

The rapid digital transformation of healthcare calls for an equally urgent ethical response. Applying the principle of justice means going beyond ensuring access—it requires structural changes that proactively address digital exclusion. From philosophical reasoning to digital literacy, co-creation, and policy alignment, we have outlined the ethical foundations and practical levers needed to promote digital health equity. Justice, understood not only as equal access but as the active correction of structural disadvantage, must become the guiding value in designing and deploying digital health systems.

## Data Availability

No datasets were generated or analysed during the current study.
